# A qualitative assessment of the acceptability of hepatitis C remote self-testing and self-sampling amongst people who use drugs in London, UK

**DOI:** 10.1186/s12879-018-3185-7

**Published:** 2018-06-19

**Authors:** Andy Guise, T. Charles Witzel, Sema Mandal, Caroline Sabin, Tim Rhodes, Anthony Nardone, Magdalena Harris

**Affiliations:** 1 London School of Hygiene and Tropical Medicine, London, UK; 2 School of Population Health and Environmental Sciences, Guy’s campus, King’s College London, Addison House, London, UK; 3 SIGMA Research, London School of Hygiene and Tropical Medicine, Tavistock Place, London, UK; 4 Public Health England, Wellington House, Waterloo Road, London, UK; 5 National Institute for Health Research (NIHR) Health Protection Research Unit (HPRU) in Blood Borne and Sexually Transmitted Infections at UCL in collaboration with Public Health England, London, UK; 6 Institute for Global Health, UCL, London, UK

**Keywords:** Hepatitis C, HCV, Remote testing, Self testing, People who use drugs, Rapid assessment, Qualitative

## Abstract

**Background:**

Hepatitis C (HCV) diagnosis and care is a major challenge for people who use illicit drugs, and is characterised by low rates of testing and treatment engagement globally. New approaches to fostering engagement are needed. We explored the acceptability of remote forms of HCV testing including self-testing and self-sampling among people who use drugs in London, UK.

**Methods:**

A qualitative rapid assessment was undertaken with people who use drugs and stakeholders in London, UK. Focus groups were held with men who have sex with men engaged in drug use, people who currently inject drugs and people who formerly injected drugs (22 participants across the 3 focus groups). Stakeholders participated in semi-structured interviews (*n* = 5). We used a thematic analysis to report significant themes in participants’ responses.

**Results:**

We report an overarching theme of ‘tension’ in how participants responded to the acceptability of remote testing. This tension is evident across four separate sub-themes we explore. First, choice and control, with some valuing the autonomy and privacy remote testing could support. Second, the ease of use of self testing linked to its immediate result and saliva sample was preferred over the delayed result from a self administered blood sample tested in a laboratory. Third, many respondents described the need to embed remote testing within a supportive care pathway. Fourth, were concerns over managing a positive result, and its different meanings, in isolation.

**Conclusions:**

The concept of remote HCV testing is acceptable to some people who use drugs in London, although tensions with lived experience of drug use and health system access limit its relevance. Future development of remote testing must respond to concerns raised in order for acceptable implementation to take place.

## Background

Hepatitis C (HCV) is a growing challenge globally. An estimated 71 million people are living with chronic infection, although only 1 in 5 people have been diagnosed [1]. In England an estimated 160,000 people live with chronic HCV infection [2]. People who inject drugs are the group most affected by HCV in the UK, with up to 90% of chronic infections linked to injecting drug use [3]. There is also a growing consensus that certain groups of men who have sex with men are at high risk of HCV infection linked to injection drug use and use of certain psychoactive substances while having sex (‘chemsex’) [4–7]. Recent surveys indicate that approximately half of people injecting drugs in the UK are aware of their HCV antibody positive status [2]. Low testing access has long been linked to treatment challenges: historically (figures for 2012) only 3% of those living with HCV accessed then available interferon-based treatment annually [8]. Treatment options for HCV have however dramatically improved through the introduction of direct acting antivirals, potentially enabling elimination of HCV as a public health threat by 2030 [9–14]. In order for this to occur radical efforts are needed to enhance diagnosis of HCV, particularly among people who inject drugs [14].

In the UK, as globally, HCV testing uptake is low and HCV treatment is difficult to access for people who use drugs [1, 8, 15–23]. Global and UK guidance on HCV testing emphasizes two stages: 1) an antibody test to establish HCV exposure, generally using either rapid diagnostic tests of blood or oral fluids, or laboratory based immunoassays; and 2) a polymerase chain reaction (PCR) test to confirm viraemic (current ongoing) infection [24, 25]. In the UK both tests are available from primary and secondary care, sexual health and genitourinary medicine clinics as well as prisons, drug treatment services and immigration centres [26, 27]. A range of individual, organizational, social and structural factors complicate access to current testing modalities in the UK, as globally: limited availability of testing; gaps in HCV knowledge; mistrust in health services and providers (linked to provider attitudes and stigma); provider reluctance to treat HCV in the context of continued drug use; and provision of services in tertiary centres which are less amenable to the needs of people who use drugs [15, 21, 28].

A potential innovation to enhance testing access is remote (or self, or home) testing [29]: testing outside clinic settings and with the person themselves implementing the test. Such an approach follows recent developments in the promotion of remote self testing for HIV [30]. Research literature on remote HIV self-testing indicates benefits in terms of convenience, privacy and managing stigma, but also highlights concerns around feasibility, acceptability and potential for harm [31]. Within the context of efforts to achieve HCV elimination, interest in remote HCV testing is emerging [29].

We distinguish two forms of remote testing for HCV: self-sampling (HCVSS) where an individual provides a sample for testing that is sent to a laboratory, which issues a result; and self-testing (HCVST) where an individual operates a simple rapid test themselves and interprets the result. In the UK remote HCV testing is currently not regulated, supported by the National Health Service nor included in associated guidelines and procedures. Self-sampling for HCV is currently available for private purchase through a number of regulated online providers. Rapid diagnostic tests intended for clinical use are also sold online and these can be used for self-testing; these tests are unregulated in the UK and are not designed for personal use, despite being marketed as self-tests. Some countries have regulated use of remote tests (e.g. USA [32]) but technologies and their uptake are nascent.

To our knowledge there has been no published investigation in the UK or globally of the acceptability of remote HCV testing for people who use drugs. Whilst experiences of remote HIV testing are informative, HCV may require different remote testing strategies to adapt to specific aspects of the testing process [29], HCV disease experience, prevailing social norms and care systems. Exploring remote HCV testing is essential to inform this potential policy priority. In response we aimed to explore the acceptability of HCV remote testing among people who use drugs in London, UK.

## Methods

We used a qualitative rapid assessment methodology that emphasises speed of research and linking assessment to action. This approach reflects a pragmatic approach to public health research with an orientation to intervention development and gaining findings adequate for policy decision making [33, 34]. Specific methods used were focus groups with people who use drugs and semi-structured interviews with key stakeholders. Data were generated in London, which has the largest proportion of people who inject drugs living with HCV in the UK [2], from January to March 2017.

For illustrative purposes only we obtained examples of HCV self sampling and HCV self testing options. The tests were not endorsed by the research team, the companies who provided them had no input on study design, data collection or analysis. The HCV self tests were inactive prototypes of an oral fluid test, similar to rapid diagnostic tests already available. The HCV self sampling kits required a blood sample to be expressed in to a vial and sent for laboratory testing.

Three focus groups were held. We sought to include people over the age of 18 with current or past experience of injecting drug use or chemsex; exclusion criteria were being younger than 18 years of age and having not previously injected drugs or engaged in chemsex. Each targeted a different drug-using population with the aim of exploring potential variation in need for HCV testing, in terms of current access to testing and how testing could be delivered including how to support this with appropriate messaging and information. Separate groups were also organised to increase comfort and reduce the impact of any stigma associated with particular identities, in that people may feel uncomfortable sharing views about HCV with people with a distinct experience to them. Groups comprised: 1. men who have sex with men engaged in chemsex; 2. people who currently inject drugs in contact with drug treatment; 3. people who formerly injected drugs. Focus group participants were recruited through partner community organisations, networks of the research team, social networking hook-up apps aimed at men who have sex with men, social media accounts of relevant support organisations and snowball sampling. Key stakeholders were interviewed in order to provide insight to current delivery of HCV testing and the policy environment for any testing innovation, as well as to triangulate with the focus group data. Key stakeholders were recruited through existing networks of the London School of Hygiene and Tropical Medicine and study partners. Focus groups and interviews were informed by similar topic guides, both structured by pre-identified domains of interest from the existing literature [15, 17–19] and through discussions with research partners. In both focus groups and interviews we initially explored views and experiences in an open and unstructured way, and then addressed specific questions on how issues such as stigma, health system logistics and psychosocial support may be important for respondents. Sample test kits were presented to inform participants of the existing technologies and to facilitate discussion; participants did not try the tests, but instead had the chance to review and inspect them. Five stakeholders (Table 1) were interviewed and 22 people participated in 3 focus groups (Table 2). Focus groups each lasted approximately 1.5 h, whilst stakeholder interviews averaged half an hour.

**Table 1:** Stakeholders interviewed

S1	S2	S3	S4	S5
Service manager from men who have sex with men and use drugs outreach organisation	Drug user network representative, living with HCV	Outreach worker, living with HCV	Activist and campaigner, living with HIV and HCV	HCV policy manager, international organisation

**Table 2:** Participants in focus groups

FG 1	FG 2	FG 3
Men who have sex with men who use drugs in ‘chemsex’ contexts	People who inject drugs in contact with drug services	People who formerly injected drugs
8 participants: all men; average age 37 (range 26–52)	6 participants: 4 men, 2 women; average age 51 (range 48–55) – 3 did not report age	8 participants: 6 men, 1 woman, 1 trans female; average age 49 (range 26–63)
3 previously had positive HCV result	3 previously had positive HCV result	3 previously had positive HCV result

We used a thematic analysis approach [35]. First level coding used an a priori framework based on the interview guide, such as perceptions of care access and responses to a positive test results, to initially organise the data. Second level coding within this organisation of the data was inductive, focusing on prominent or significant aspects of respondents’ accounts and oriented to developing a grounded account of how participants spoke about HCV testing [35]. Through the analysis we compared data from the different focus groups and interviews in order to understand if and how specific experiences of views on HCV testing varied according to the themes identified.

The research presented was undertaken with the National Institute for Health Research Health Protection Research Unit (NIHR HPRU) in Blood Borne and Sexually Transmitted Infections at University College London. The study obtained ethical approval from the London School of Hygiene and Tropical Medicine (ref 12,054). All participants gave informed written consent. Each participant in the focus groups received 40 GBP to cover traveling expenses and time.

## Results

We developed an overarching theme of ‘tension’ during the analysis to explore the acceptability of remote testing for people who use drugs. Our use of ‘tension’ as an organising theme allowing expression of how responses to the tests were sometimes contradictory and ambivalent, or brought out issues that were described as not easily resolved. We identify tension in how enthusiasm for the principle of remote testing was reported alongside flaws in the promise of remote testing, and also in how its principle was seen as challenging to implement. For example, individuals might express excitement for remote HCV testing but also recognise inherent challenges for people who use drugs:

*I can certainly see the benefit in sitting at home in private and doing it, I reckon a lot of people would be in to that for all sorts of reasons. But it’s… I don't know, it’s possible that maybe those same people are the people that would be most worried if they got a positive result. (S4)*

Or accepting the principle of the tests, but alongside concern at health system capacity to sustain them:

*Pushing for testing is great, but I think that the fact that even with the new drugs and the rationing… you’ve still only got about 7% of those infected accessing treatment each year … you push testing and the chances are they won’t get treatment for years. I think you really have to weigh that up carefully. (FG3)*

The over-arching theme of tension reflects long standing concerns around implementation of novel interventions [36] and accounts for the sometimes competing priorities and pressures that link the separate themes we report: choice and control, ease of use, embedding testing in appropriate care pathways, and managing an uncertain result. In exploring each theme we draw attention to these tensions and explore the implications of this in the discussion.

### Choice and control

Many participants considered the potential choice and autonomy over testing that a remote option might bring as important:

*I quite like the idea actually that you can do this without the big faff around with your clinic, without them taking control of it all, that you take control of it and you take it home (S4)*

The importance of autonomy was particularly evident in response to a desire for privacy and to address stigma; reported in the lives of participants both in relation to drug use and HCV. Remote testing provided a way to manage this stigma, through removing the need to disclose to health care providers:

*We can talk about de-stigmatisation and things, these things take time and they take resources, etc. etc. and in the meantime something like this can easily bridge that gap I think (S1)*

A sense of remote testing as an important choice and focus for control was not universal, with some participants cautioning on the potential for particularly vulnerable people to enact this ‘choice’ and whether it was needed (as we discuss below), and also some questioning whether this was a meaningful choice:

*I don’t see the point… because once you do the test, and you find out…so you have to run to the doctor. So, why don’t you go in the first place? (FG2)*

### Ease of use

Tensions become particularly evident when participants explored the practicality of implementing a test themselves. The different types of tests generated distinct responses on their ease of use. The self-sampling kit required a blood sample by pricking a finger and then squeezing blood in to a vial approximately half a centimetre wide. This was perceived by some as arduous, complicated and painful to collect the required sample.

*I would feel, God, there’s so many ways this could go wrong. And also, even though there’s lots of instructions, I think it’s the crucial moment, that point of getting the… spiking your finger and getting the blood out (FG1)*

Such complications led to suggestions that the test would be impractical for those experiencing extreme hardship:

*This wouldn't work at all. There's just too much going on here on the second one [self sample] for somebody who's homeless. Just wouldn't work at all. (S2)*

Although the ‘awkward’ nature of the self-test was offset for some who felt more ‘confidence’ in the result it would give.

The self-testing option – in essence a rapid diagnostic test – was in comparison seen as beneficial in regard to the immediacy of the result, simplicity of operation and use of saliva rather than blood. It was felt to cater to a wide range of abilities, something important for a target population characterised by marginalisation.

*I think it’s the accessibility of the test is what I enjoyed. The first one, and knowing that I would get my result immediately. Almost immediately.* (FG2)

*I suppose I think being saliva would probably be more comfortable because I know myself, I get really cautious around blood.* (S3)

### Embedding remote testing in appropriate care pathways

There were widespread concerns about ensuring appropriate care pathways, linked to fears people may be lost to care. Many participants stated that remote testing should be linked to education, support and follow-up care: ‘t*he care pathway is so key to this and it has to be clear’ (S1).* A recurring response was for the need for test access to be linked to engagement with a provider or other expert who could give information and provide support:

*I don’t think it’s a solitary process this. I think you need that support and that safety net in place, whether it be for a peer or… I think what it’s got is portability so you can go into a hostel with it and do the test on that day. (S3)*

Other responses suggested test access should be supported, whether through homeless hostels or specific outreach processes – in effect meaning that remote testing technologies would be delivered as community based testing – or be offered within clinic settings linked to clear information and support:

*I think the priority would be hostels… I think it's the best place to pick up the guys that are most at risk. (S2)*

*You initially have a discussion with the Consultant in the clinic and they explain everything, and then you can get them on order to your house every three months, or whatever, with the understanding that if you do get a positive result, or if you want to go back in, you can go and discuss it with them. (FG1)*

Online ordering was one suggested medium for remote testing access. This was perceived as convenient, especially amongst those in the men who have sex with men focus group, and to create a threshold to ensure people had sufficient understanding, in response to the delineation of sufficient agency:

*I also don’t want to disempower half the population who are quite capable…I suppose if you are actively, you have the abilities to go online and do that research, maybe that’s the factor where you show that you’ve got awareness and you’re sure about your accountability. (S3)*

However, as illustrated by this stakeholder who was also a representative of a drug user network, an online approach could exclude some, particularly among those who had limited access to or interest in online services:

*The only real drug users that are online are more the psychoactive or psychoactives. Opiate users, crack users aren’t online. No. Completely excluded. (S2)*

Care pathway concerns were not universal. A self sampling option was seen by some to be beneficial in that it offered some basic linkage to specialist support, in terms of a phone call to provide the result:

*I’m sending it to a lab to be tested and I’m able to sit at home and do it all in the privacy of my own home and then send it away and then someone is going to ring me up afterwards and say, hopefully, be a bit thoughtful in how they tell me positive or negative. I think that’s why I would prefer this one. (S4)*

Remote testing for some men who have sex with men was perceived to be of less overall utility in the context of many in this group already having strong health system access. There were reports of attending sexual health clinics frequently for routine HIV and STI testing, with the assumption that testing for HCV was embedded within their sexual health care pathways.

*Under what circumstances would I select Hep C for a home test, if I’ve still got to go to the clinic for all my other tests? You know, and is there some specific reason for me to do it? … why bother to go through all this risk and get, you know, all the anxiety I know I would have with a positive reaction, when I’ve got to go to the clinic in three months anyway for all my other tests. (FG1)*

Concerns extended to whether the health system could provide a suitable care pathway. Sometimes grounded in peoples’ own experiences of seeking HCV treatment, there were complaints about the available infrastructure that led to questioning the ethics of remote testing under such conditions of scarcity:

*Part of me wants to actually say “What?” I’m on a waiting list waiting for [HCV] treatment. You get awareness. Is the infrastructure in place for dealing with an increase in people? (S3)*

### An unsupported and uncertain diagnosis

Participants raised concerns about how a positive test result might be understood and experienced, particularly if done in isolation. This was a barrier to the acceptability of remote testing and undermined its potential as a viable choice for many. Many participants suggested a positive HCV result could cause ‘stress’, a ‘bad reaction’, ‘anxiety’ (FG1), ‘panic’ (FG2), be ‘serious relapse material’, ‘traumatic’ (FG 3), especially in the context of having no direct link to care or support:

*If you get a positive result and you’ve got no information, that could be really scary…panic, panic, panic. (FG3)*

Accounts differentiated between groups and individuals in the potential for managing a test result, distinguishing between those with the knowledge or social support to manage the choice remote testing offered, and those who may struggle: those experiencing shame, with mental illness or under the influence of drugs:

*Maybe it should be for people who actually know what they’re dealing with, who know what that is, and they want just to test themselves. (FG1)*

*I don’t think it’s a great tool for somebody who’s at the front line, cold face, bang on addiction. I think that it has to be higher-up or further along the path. (S3)*

Potential for alarm was compounded by confusion about the meaning of a positive result. There was a widespread, though not universal, lack of understanding of HCV testing requiring two stages, of initial serological tests to assess exposure, and then a confirmatory PCR test to assess presence of the virus. The self test option for example shows exposure to the virus by testing for antibodies, but not if the virus is currently active or has been naturally cleared (which requires a PCR test). In response, remote testing was seen as potentially ‘misleading’ (FG 1) with others highlighting the challenge of understanding the testing process:

*I’ve been in some discussions with some people. There’ve been arguments between drug users when someone said they’re Hep C but they don’t have it, and somebody trying to get their head around that. If you haven’t got the information to back it up, when you explain that to someone it’s really hard to digest. (S3)*

Confusion about the potential meaning of a test result can be linked to the limited information in the test packs. Neither test option explained the test result meaning with reference to HCV antibodies or the virus itself. However, one of the tests was a prototype, and so accompanying information hadn’t been tailored for remote use. Participants made requests for clarifying information to be included with the tests to address the uncertainty of a diagnosis and also on follow-up care:

*We should have at least an information on this, you would need some help straight away. If you get a positive result and you’ve got no information, that could be really scary (FG3)*

## Discussion

Using a rapid assessment approach, we qualitatively explored the acceptability of remote HCV testing for people who use drugs in London. Our results show that remote HCV testing generates responses characterised by tension between competing pressures. There is tension in how participants valued the potential for choice from remote testing, whilst the enactment of such principles is considered not feasible for some given experiences of drug dependency, material hardship and unsupportive health systems. Further tension comes in how a principle benefit of remote testing is to potentially separate testing from stigmatising and remote care systems, and yet any positive result nonetheless requires a supportive care system.

These tensions we understand as reflecting in part the specific complexities of diagnosing and treating HCV but also the social contexts and experiences for many people who use drugs: experiences of economic and social marginalisation, as well as mistrust of care systems grounded in stigma and abuse [21]. Given these tensions, and concerns about receiving a test result in isolation or that novel strategies are potentially less needed for men who have sex with men using drugs reflecting potentially strong health system access, the current remote testing technologies may only be of utility and value for some people who use drugs within the current social and health system contexts. This could include those not in contact with drug services (where HCV testing should be widely available) but with an understanding of the HCV testing pathway and ability to access and negotiate confirmatory testing and follow-on care. This demographic may comprise people who formerly injected drugs, medically literate but reticent to disclose past risk practices in order to access a HCV test. Considering this potentially limited relevance of remote testing we recommend that remote testing be modified and supportive health and social support systems developed to enable broader acceptability and encourage effective use.

In order to ensure broader acceptability we recommend that developers of remote HCV testing technologies consult with local groups of people who use drugs and service providers to ensure accompanying information provided is relevant and accessible. Clarity is required around what the test is testing for and – if for antibody only – why a confirmatory test is required and how to access it. A central concern among participants was the potential for harm if a self-testing test result was obtained in isolation (we note how the same concerns have long been raised with HIV self testing, although with little evidence of such harms [31]). This was compounded by confusion between the meaning of test results [37, 38] and barriers to accessing confirmatory and follow-on care. There is therefore a need for remote testing to be developed and employed in tandem with comprehensive supportive care pathways [39, 40], and for this detail to be included in accompanying information with tests.

Participants indicated a desire for remote testing to be integrated into clinic level care, or outreach and hostel support as part of a supported care pathway, rather than offered in isolation [41]. As suggested, outreach workers could provide support and information, and then distribute tests and collect samples for testing, or be available in the event of a positive test result in order to aid interpretation and understanding (whether in person, or over the phone). Such a pathway could also enable referrals in to appropriate care. Exploration and development of these pathways would mitigate some of the potential for harms noted, regarding remote testing confusion, lack of support and associated distress. Pre and post-test engagement aligns with recommendations for community based HCV testing, as per current UK and World Health Organisation guidelines [2, 24]. Developing such a hybrid model of supported remote testing may increase acceptability.

Participants were concerned about the relevance of a test in a context of uncertain HCV treatment access, with people who use drugs facing barriers to treatment access both in the UK and globally [1, 21, 42]. As noted by participants, remote testing does not address or alleviate the broader health service and structural issues which complicate access to HCV treatment for people who use drugs [21]. In tandem with any efforts to develop testing technologies and signposting to appropriate care pathways, is the need for action on the social and structural constraints to HCV treatment access, including, for example, uneven treatment availability; geographical access and transport affordability; criminalisation and incarceration; discrimination and stigma within healthcare settings [43].

An overall implication of our findings is to suggest that HIV remote testing models that prove acceptable for some groups [44] may not be easily replicated for HCV for people who use drugs. A model of remote testing as currently formulated may have relevance for certain populations who are vulnerable to HCV or within a hybrid model of testing within community services. However, specific considerations of the potential for confusion around testing and linked awareness of HCV as well as treatment access barriers [28, 45] require a tailored approach to HCV. The utility of self-sampling and self-testing is likely to be context, population and infection specific and so such strategies for HCV will need further development and evaluation.

These results should be understood within the scope of the rapid assessment methodology used. Our analysis should be interpreted cautiously reflecting the limited time we had to collect data. More research is needed to elaborate and refine our findings and explore their transferability to other settings. In particular, the geographic focus and limit to London suggests any future study should be expanded to other urban areas, as well as rural settings within the UK to understand varying need. An additional priority would be more in-depth study of usability, in particular more fully including service users within design and development of the technologies. However, given the limited evidence for the potential use of remote HCV testing these qualitative results give insight in to the perceptions of potential users of remote testing and so are an important basis for initial policy deliberations.

## Conclusions

Rapid qualitative assessment in London of remote HCV testing for people who use drugs shows potential acceptability alongside many concerns. Current technologies may only be suitable for a medically literate population with access to follow-on care and linked support, which may not be the majority of people who use drugs. In order to be ethically employed among broader populations, including those most at risk of HCV acquisition, developers of remote testing technologies must work with people who use drugs and service providers to ensure the inclusion of accessible information and linkage to care and consider hybrid models using self sampling or self testing in existing provider services. While exploratory, we envisage that our findings will help inform policy discussions and health care delivery as well as debate amongst potential service users in the UK and elsewhere as this emergent technology is explored.

## Abbreviation

HCV: Hepatitis C
